# Effects of nozzle diameter and fiber volume fraction on tensile properties of FDM-printed CCF/PLA composites

**DOI:** 10.1371/journal.pone.0356177

**Published:** 2026-08-19

**Authors:** Tao Zhao, Xin Dong, Hengfa Wu, Yang Gao, Ting Lei, Fei Zheng

**Affiliations:** 1 Ningxia Engineering Research Center for Hybrid Manufacturing System, College of Mechatronic Engineering, North Minzu University, Yinchuan, China; 2 School of Electrical and Information Engineering, North Minzu University, Yinchuan, China; 3 Basic Experimental Teaching and Engineering Training Center, North Minzu University, Yinchuan, China; King Mongkut's University of Technology North Bangkok, THAILAND

## Abstract

This study investigates the effects of nozzle diameter and continuous carbon fiber (CCF) volume fraction on the tensile properties and failure mechanisms of fused deposition modeling (FDM) printed CCF/polylactic acid (PLA) composites. Two nozzle sizes (1.0 mm and 0.8 mm) and four CCF volume fractions (0%, 20%, 30%, 50%) were adopted to prepare six groups of specimens for uniaxial tensile tests and field emission scanning electron microscopy (FE-SEM) characterization. The results indicate that CCF-reinforced specimens exhibit nearly no plastic deformation and fail mainly by fiber fracture, while neat PLA shows obvious plastic deformation and tensile fracture. For the 1.0 mm nozzle, the tensile strengths reach 36.4 MPa, 34.8 MPa and 19.8 MPa at CCF volume fractions of 50%, 30% and 20%, respectively. The tensile strength is 16.9 MPa for specimens with 50% CCF printed using the 0.8 mm nozzle. Larger nozzle diameter effectively improves tensile strength, which increases approximately linearly with rising CCF content. Microscopic observations reveal that the 1.0 mm nozzle ensures uniform fiber distribution and strong fiber-matrix interfacial bonding. Considering mechanical performance and cost, 30% is determined as the optimal CCF volume fraction. With the merits of light weight, high strength and environmental friendliness, the composites are suitable for aerospace lightweight parts, automotive components, civil engineering load-bearing structures, customized tooling and medical devices.

## 1. Introduction

As an emerging rapid prototyping technology, 3D printing has found widespread application across diverse fields [1]. Fused Deposition Modeling (FDM), a prominent form of 3D printing, is widely used in engineering applications thanks to its low cost and operational simplicity [2]. While 3D printing offers numerous advantages unmatched by conventional manufacturing processes, most 3D-printed plastic structural parts remain confined to the conceptual prototype stage. This limitation stems largely from the inferior strength and performance of 3D-printed plastic components compared to functional parts, precluding their use as practical load-bearing members [3]. FDM 3D printing exhibits distinctive advantages in the field of CCF composite manufacturing. Its core superiority lies in the digitally precise placement of fibers, the achievement of lightweight and high-strength structures, and the fabrication of complex geometries [4,5]. FDM features low cost and excellent process flexibility. It can fulfill the full manufacturing workflow from prototyping to small-batch production, and has become one of the most widely adopted additive manufacturing technologies [6,7]. Benefiting from high design freedom, tunable product performance, simple fabrication procedures and superior economic efficiency, FDM has evolved into a core manufacturing technique for the research and industrial application of continuous carbon fiber (CCF) composites [8]. Numerous studies have demonstrated that FDM is highly suitable for manufacturing lightweight, high-strength and customized components. This technology broadens the application scope of carbon fiber composites and promotes the large-scale popularization of such high-performance materials beyond high-end customized fields [9]. Furthermore, FDM exhibits great compatibility with various reinforcing fillers including glass fibers and plant-based powders, showing promising prospects for the development of diverse polymer composite systems [10].

CCF-reinforced polymer demonstrates significant potential for overcoming the inherent mechanical limitations of parts fabricated by FDM [11]. A substantial body of research has successfully enhanced the mechanical performance of 3D-printed components through the strategic incorporation of reinforcing phases, including continuous or discontinuous fibers and particulate fillers. This reinforcement strategy is well established in composite manufacturing, where fiber-reinforced polymer (FRP) systems are deliberately engineered to capitalize on the exceptional specific strength and stiffness of carbon fibers [12]. When embedded within thermoplastic matrices—such as PLA, a biodegradable and environmentally benign polymer—CCF enables the fabrication of high-performance components tailored for demanding applications in aerospace, automotive, and civil infrastructure sectors [13]. Moreover, the synergy between PLA’s sustainability profile and the structural advantages conferred by continuous fiber reinforcement aligns closely with global imperatives for eco-efficient advanced manufacturing [14]. Empirical evidence consistently indicates that continuous fiber integration yields substantial improvements in tensile strength and elastic modulus—key metrics for structural integrity—thereby positioning continuous fiber-reinforced polymer FDM as a compelling pathway toward next-generation lightweight composite production [15].

Li et al. developed a novel continuous fiber-reinforced 3D printing technique integrated with an optimized toolpath planning strategy. This approach involved a structural modification of the extrusion nozzle in a conventional FDM system, enabling the successful fabrication of standardized CCF/PLA components. The mechanical and thermomechanical properties of CCF-reinforced composites were obtained via tensile tests, three-point bending tests, and dynamic mechanical analysis [16]. Due to the poor interfacial bonding between carbon fibers and PLA, which severely deteriorates the mechanical properties of the composites, a surface preparation method for carbon fibers was proposed, and the carbon fibers were modified using a PLA dispersion slurry [17].

Enhancing the ductility of 3D-printed continuous fiber composites, together with their tensile strength, compressive strength, and fatigue resistance, will open new prospects for their application in high-performance engineering [18]. Dou et al. systematically investigated the tensile mechanical behavior of CCF/PLA composites fabricated via FDM, by varying key process parameters including layer height, extrusion width, nozzle temperature, and print speed. Their findings demonstrated that the relative fiber content—defined as the volumetric or areal fiber fraction within the printed laminate—exerts a dominant influence on the overall mechanical performance. The mechanical properties of neat PLA, carbon fiber-reinforced PLA, and modified carbon fiber-reinforced PLA were systematically compared. Experimental results reveal that the tensile strength of carbon fiber-reinforced PLA is significantly enhanced compared to that of neat PLA. Moreover, surface modification of carbon fibers is found to further improve the flexural strength of the resulting composites. Calvo et al. further examined the compressive and flexural responses of nylon-based composites reinforced with either carbon or glass fibers, explicitly correlating these properties with the fiber volume fraction of the reinforcing phase [19].

Although nanoparticle or fiber-reinforced PLA composites exhibit enhanced mechanical performance and additional functional benefits, further systematic optimization—particularly of reinforcement architecture, interfacial design, and FDM process parameters—remains essential to fully realize their potential in advanced additive manufacturing applications. The influence of printing parameters on the mechanical integrity of FDM-fabricated components is well established [20,21]. Kechagias et al. quantitatively evaluated how layer height and nozzle temperature affect tensile strength, flexural stress, and impact toughness. Using a combined approach integrating analysis of variance and numerical simulation, they identified an optimal configuration of 0.2 mm layer height and 215 °C nozzle temperature, which maximized the overall mechanical performance of printed PLA specimens [22]. Elmrabet et al. systematically examined the effect of infill density on compressive and tensile behavior, the optimum compressive modulus and tensile strength were 2.26 GPa and 54.20 MPa at a 100% infill ratio [23]. Lou et al investigated multiple process variables—including layer height, wall thickness, extrusion temperature, and print speed—in thermoplastic polyurethane (TPU) parts, confirming that these parameters exert statistically significant and interdependent effects on mechanical outcomes [24]. Collectively, these studies underscore a growing consensus in the literature: layer height, nozzle temperature, and print speed are critical, interrelated determinants of mechanical performance across diverse polymer systems and FDM platforms.

Existing research on extrusion-based additive manufacturing polymer composites covers multiple mainstream research directions. For sustainable structural fabrication, machine learning optimized 3D printing technology has been adopted to prepare agave fiber-reinforced PLA composites, and strategically interleaved continuous sisal fiber core structures have been proven effective in boosting the mechanical performance of PLA engineering parts, yet these studies only focused on natural plant fibers without discussing continuous carbon fiber systems and the coupled influence of nozzle dimensions and fiber volume fraction on tensile properties [25,26]. In terms of recycled material manufacturing, fused deposition modeling has been utilized to fabricate recycled polypropylene filled with aluminum powder, revealing the significant regulation effect of filler loading on composite mechanical behaviors, though the thermoplastic matrix and reinforcement type differ greatly from continuous carbon fiber-reinforced PLA [27]. Advanced multi-degree-of-freedom additive manufacturing technologies including 5D and 3–6D printing have also been systematically reviewed; such multi-axis equipment can deposit fibers along stress trajectories to improve structural integrity, but the high equipment cost limits its large-scale application in conventional FDM production lines [28,29]. Meanwhile, studies on biomedical 3D printed polymer-ceramic composites further confirm the synergistic strengthening mechanism of rigid fillers within polymer matrices: akermanite and zirconia co-modified PMMA bone cement composites and bionic structured akermanite/PMMA/zirconia 3D printed implants both achieve balanced mechanical and biological performances via adjusting filler content, which provides theoretical reference for the fiber reinforcement strategy of thermoplastic matrix composites, while relevant investigations rarely take nozzle diameter as a core variable for quantitative mechanical analysis [30,31].

The mechanical test results reveal that the compressive and flexural performance of FDM-printed composites is progressively improved with the increase in CCF volume fraction, and an appropriate elevation in fiber content can effectively enhance their tensile performance within a reasonable range. In recent years, numerous studies have investigated the effects of process parameters and fiber content on the mechanical characteristics of fiber-reinforced polymer composites. Existing literature has demonstrated that a moderate increase in CCF volume fraction optimizes the tensile, compressive, and flexural properties of printed components, whereas excessive fiber loading induces fiber aggregation, insufficient matrix infiltration, and increased internal porosity. These defects severely degrade fiber-matrix interfacial bonding strength and hinder the further improvement of composite mechanical performance. Most previous studies have focused primarily on single-factor modulation, including layer height, nozzle temperature, printing speed, and infill density, or independently explored the correlation between fiber volume fraction and structural performance. Several studies have proposed an applicable nozzle diameter range of 0.4–1.0 mm for CCF/PLA fabrication and confirmed that small-diameter nozzles can improve fiber alignment and reduce internal defects, though ultra-small nozzles are prone to fiber damage and nozzle clogging. Nevertheless, most available investigations adopt a fixed nozzle diameter to analyze the performance variation of composites with different fiber contents, or explore nozzle diameter effects under a single fiber volume fraction condition. To date, systematic research concerning the synergistic coupling effects of nozzle diameter and CCF volume fraction on the mechanical behavior of 3D-printed CCF-reinforced polymer composites remains limited. In particular, the interactive mechanisms between nozzle size and ultra-high CCF volume fraction (up to 50%) in modulating microstructure evolution, interfacial adhesion, and macroscopic failure modes are still unclear, representing a critical research gap in this field.

Accordingly, the present study systematically investigates two typical nozzle diameters (1.0 mm and 0.8 mm) combined with four CCF volume fractions (0%, 20%, 30%, and 50%), covering low, medium, high, and ultra-high fiber content levels. Axial tensile tests are conducted with six replicated specimens for each parameter set to guarantee experimental repeatability and data reliability. The composite failure mechanisms are comprehensively explored by combining macroscopic fracture morphology observation and high-resolution microscopic characterization. Field emission scanning electron microscopy (FE-SEM) is employed to characterize the cross-sectional microstructural features of printed specimens, aiming to clarify the underlying strengthening mechanisms of nozzle diameter and CCF content on tensile performance. Furthermore, the relative fiber content and fiber-matrix interfacial adhesion strength are quantitatively analyzed, and the tensile failure modes under different parameter combinations are systematically summarized. This work innovatively clarifies the coupling effect between nozzle diameter and CCF volume fraction, fills the research gap regarding parameter interaction mechanisms for high-fiber-content CCF/PLA printed composites, and provides a feasible theoretical and technical reference for the precision manufacturing and performance optimization of high-strength lightweight composite components via FDM 3D printing.

## 2. Printing materials

**Fig 1:**
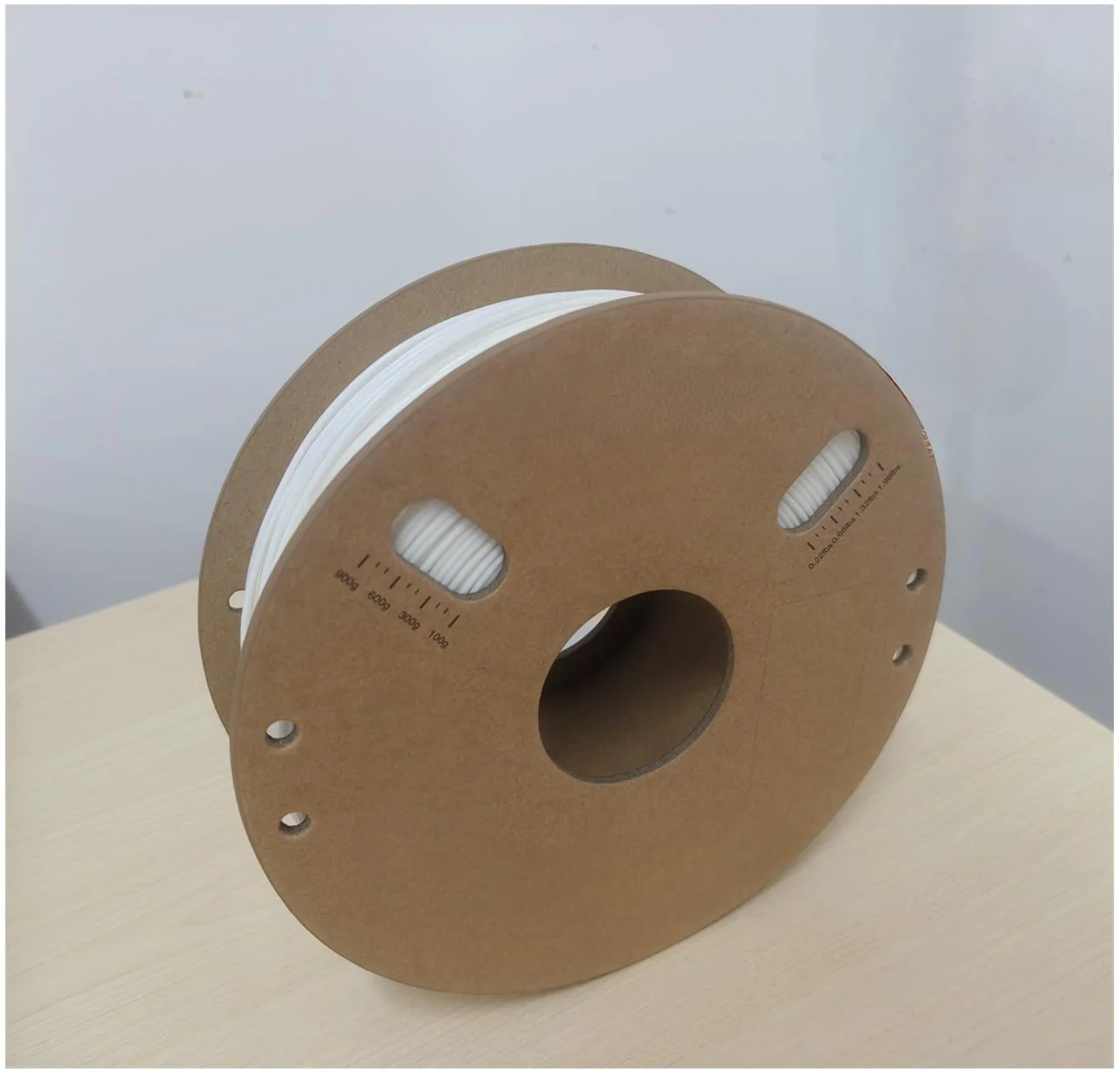
Hyper PLA filament. All photographs are original self-taken images of commercially purchased equipment and consumables, published under a CC-BY 4.0 license.

**Fig 2:**
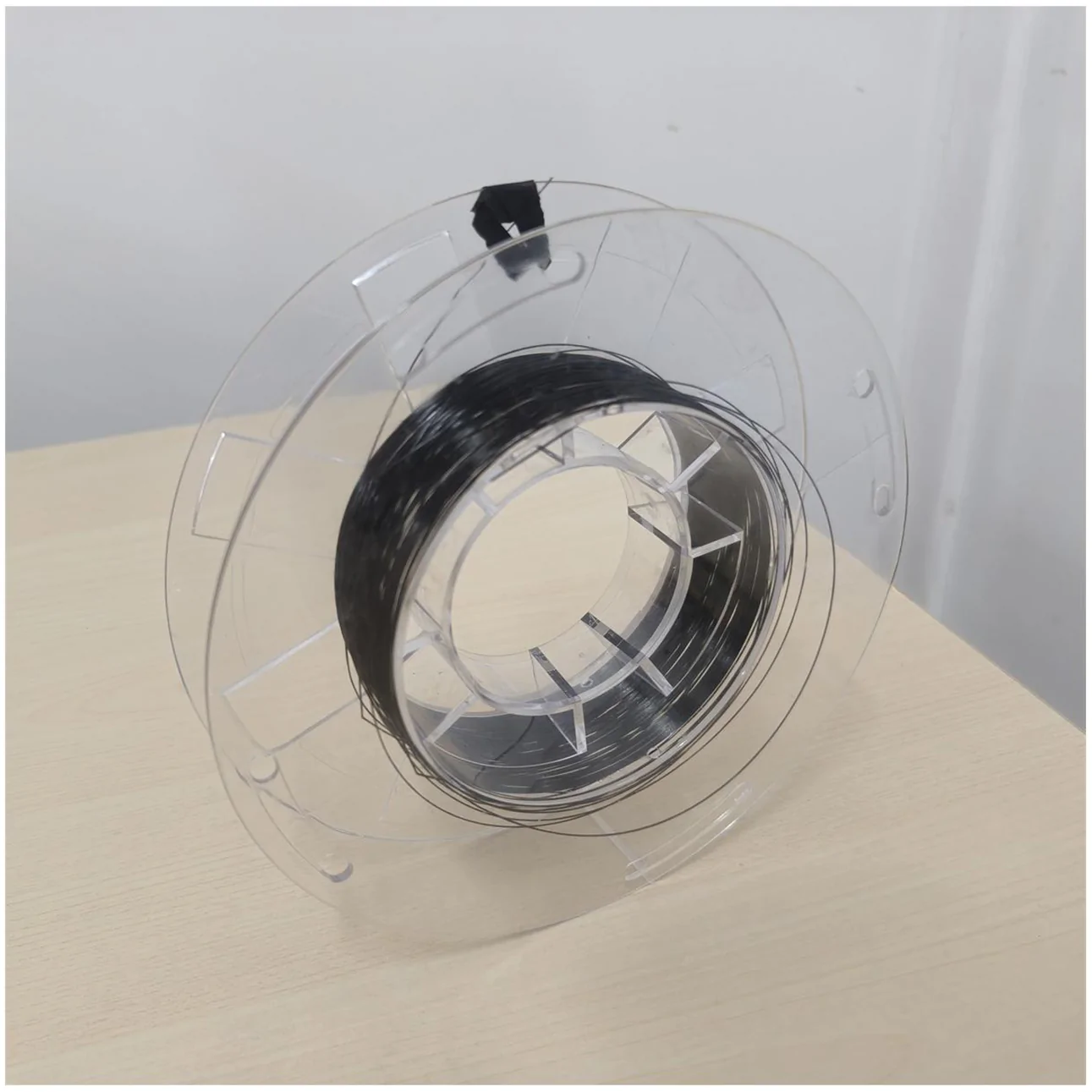
HTS40-1K CCF.

PLA filament with a tensile strength of 63 MPa and a tensile modulus of 1.9 GPa was used as the polymer matrix for the composites. 1K CCF tows, consisting of 1000 monofilaments, with a tensile strength of 3500 MPa and a tensile modulus of 230 GPa, were employed as the reinforcement. In this study, PLA was used as the matrix material, while CCF served as the reinforcement phase. PLA is a biodegradable thermoplastic polymer derived from renewable resources such as corn and sweet potato, and has been widely used in 3D printing, food packaging, and medical applications. As illustrated in Fig 1, in this study, Creality Hyper Series Hyper PLA WHITE filament was adopted, which is a 1.75 mm-diameter, 1 kg net weight white PLA 3D printing filament optimized for high-speed printing, supporting a printing speed range of 30–600 mm/s, with a recommended nozzle temperature of 190–230 °C, a bed temperature of 25–60 °C, and requiring the fan to be turned on during printing. The spool is made of recycled materials, and the filament complies with RoHS and REACH environmental standards, achieving a balance between high-speed printing performance and environmental friendliness. As a reinforcing phase, CCF is a lightweight engineering material with high strength, high stiffness, low coefficient of thermal expansion, high temperature resistance, and excellent chemical resistance. These characteristics enable the widespread application of CCF in aerospace, construction and racing industries [3]. As shown in Fig 2, Toho Tenax HTS40-1K CCF is used in this work. Surface treatment can improve the interfacial adhesion between CCF and the resin matrix, while protecting the fibers via favorable wettability and processability.

## 3. CCF 3D printer modification and experimental equipment

**Fig 3:**
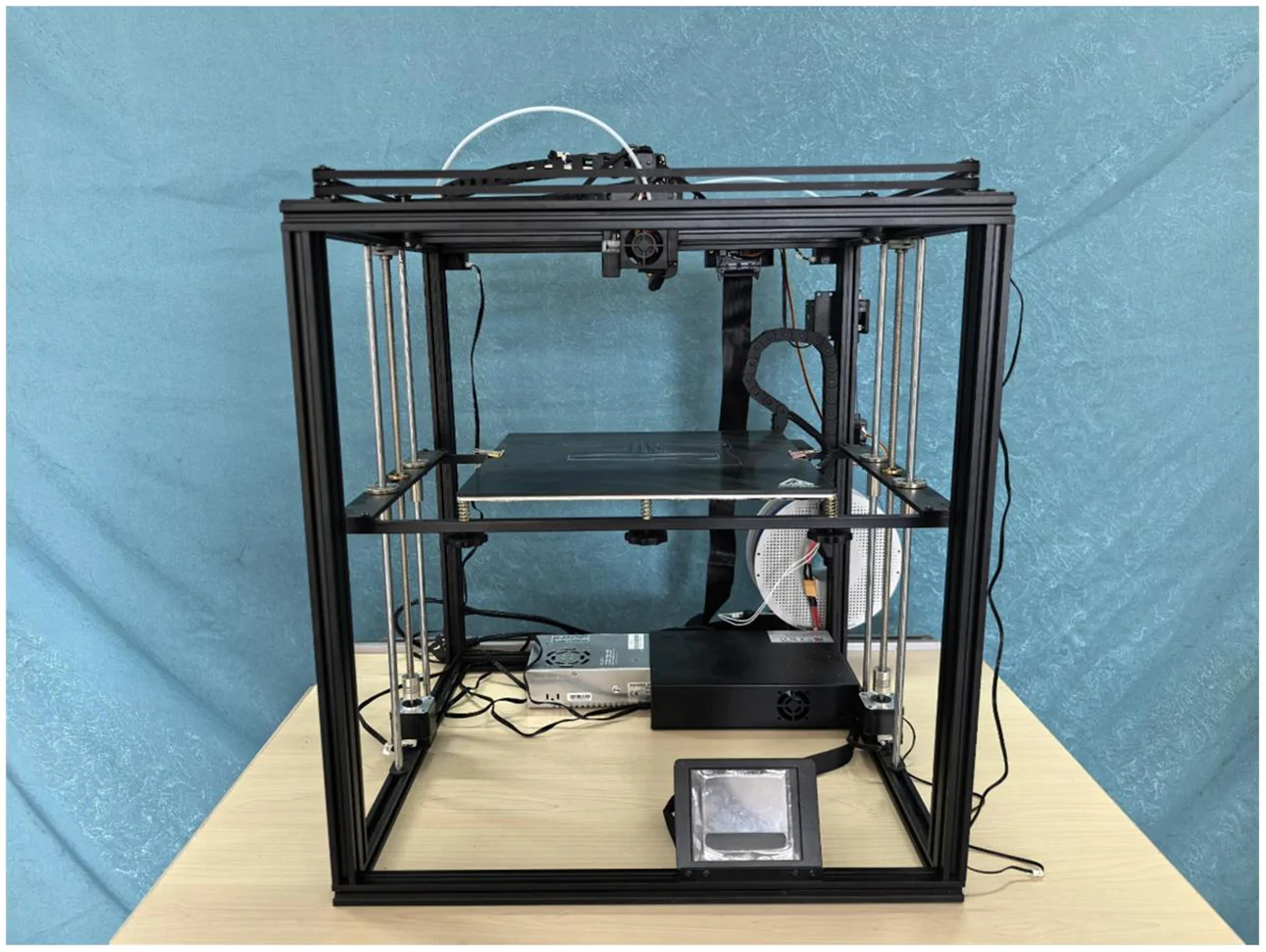
The modified 3D printer.

In this study, a desktop-grade TRONXY 3D printer is used and further modified accordingly, as shown in Fig 3.

**Fig 4:**
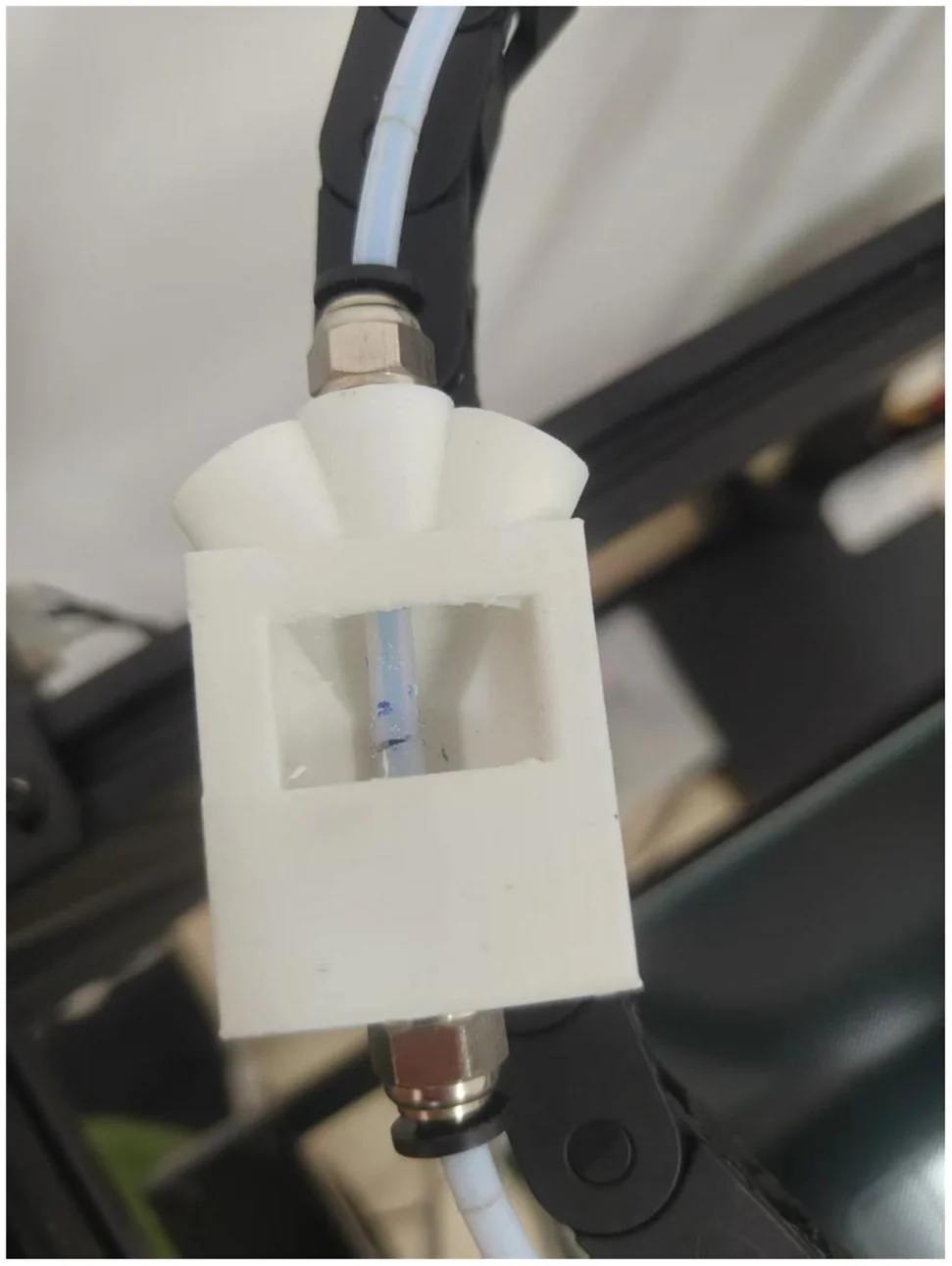
Tee connector.

**Fig 5:**
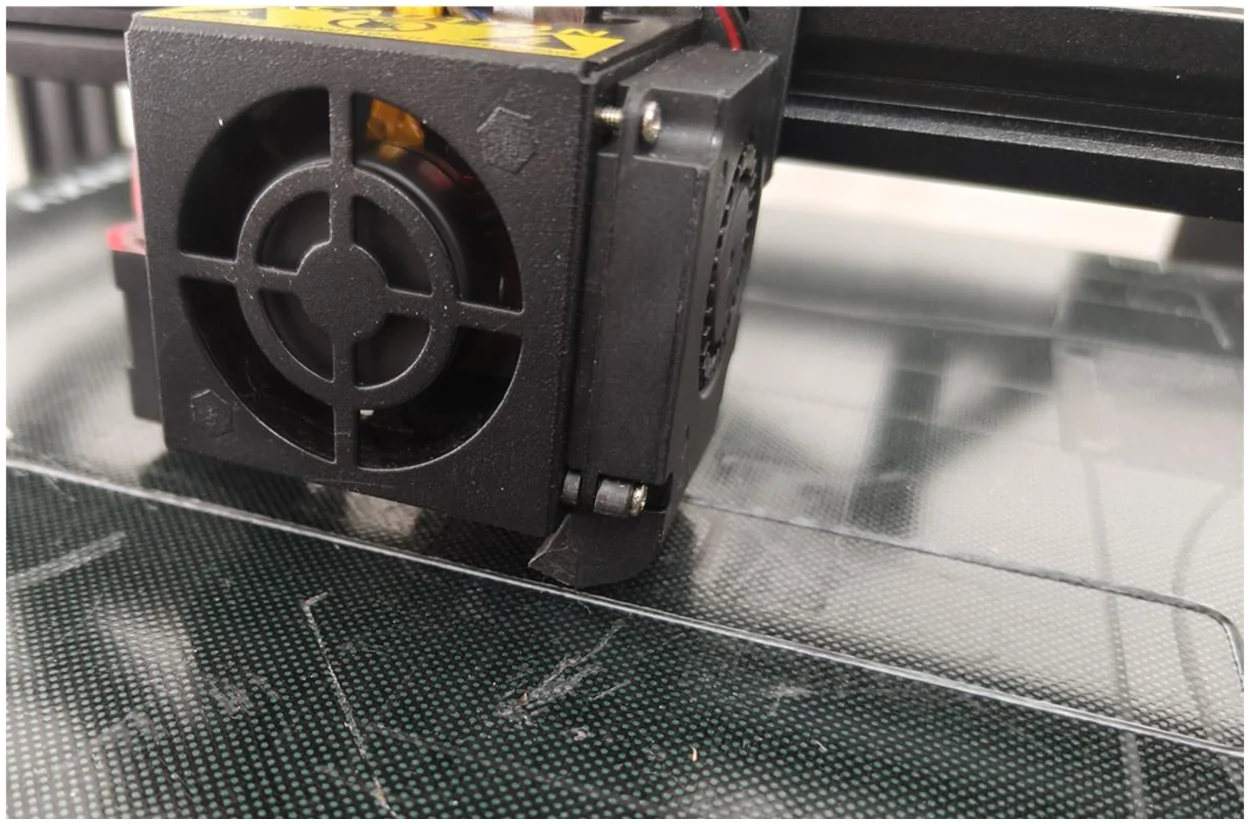
CCF/PLA Composites printing process.

Two modifications to the 3D printer are mainly concentrated on the nozzle and feeding tubes. The original nozzle of 0.4 mm is replaced with customized nozzles of 0.8 mm and 1.0 mm. A Tee connector is employed to link the two feeding tubes in Fig 4, where PLA filament is fed through the central channel, and CCF is supplied through the two side channels. In the 3D printing process, PLA is heated to a viscous flow state and fully impregnates the CCF, ensuring good interfacial bonding. Under internal pressure, the semi-liquid thermoplastic and CCF are extruded through the nozzle and deposited onto the build platform. The printing procedure is illustrated in Fig 5.

**Fig 6:**
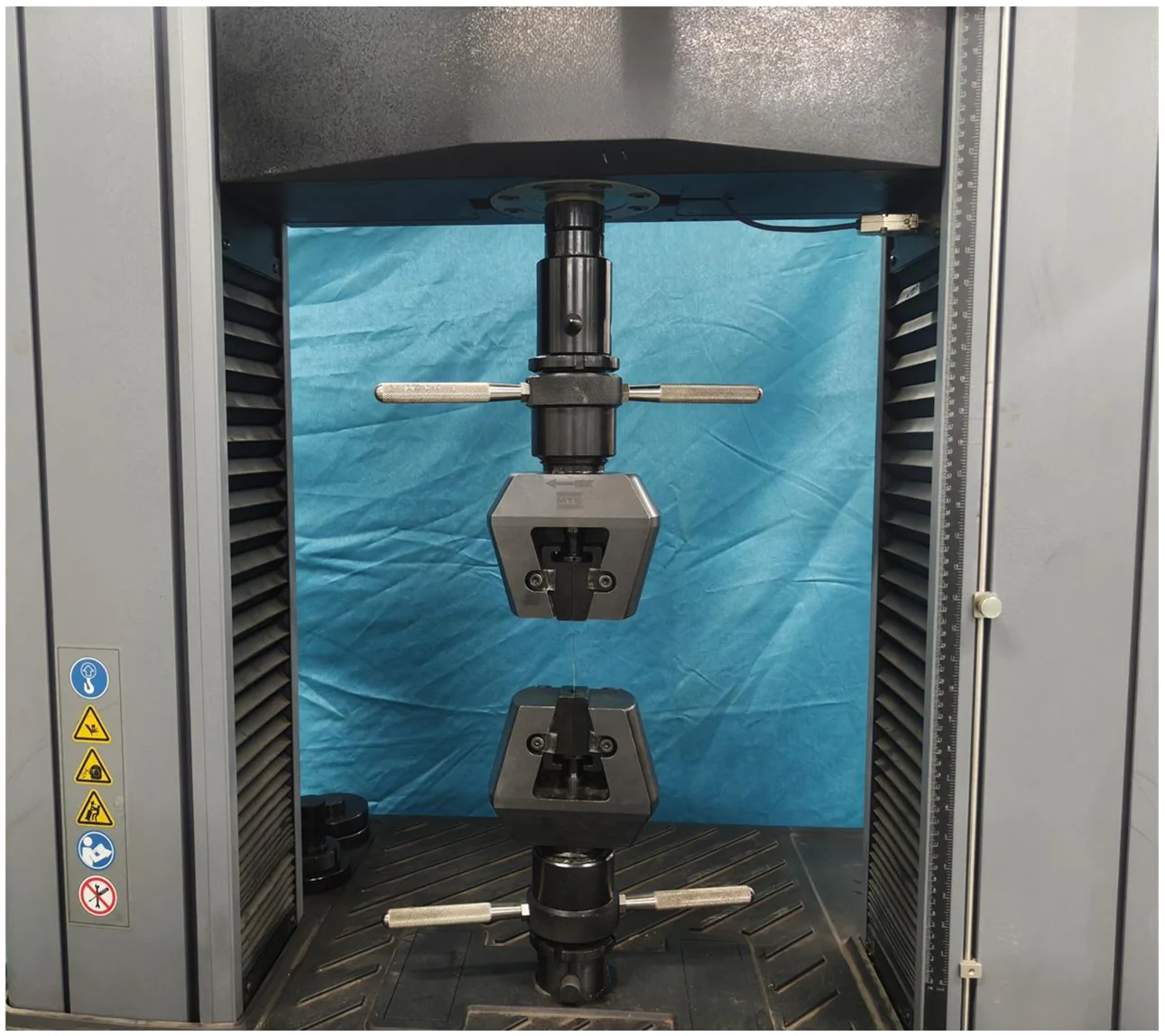
MTS universal testing machine. All photographs are original self-taken images of commercially purchased equipment and consumables, published under a CC-BY 4.0 license.

**Fig 7:**
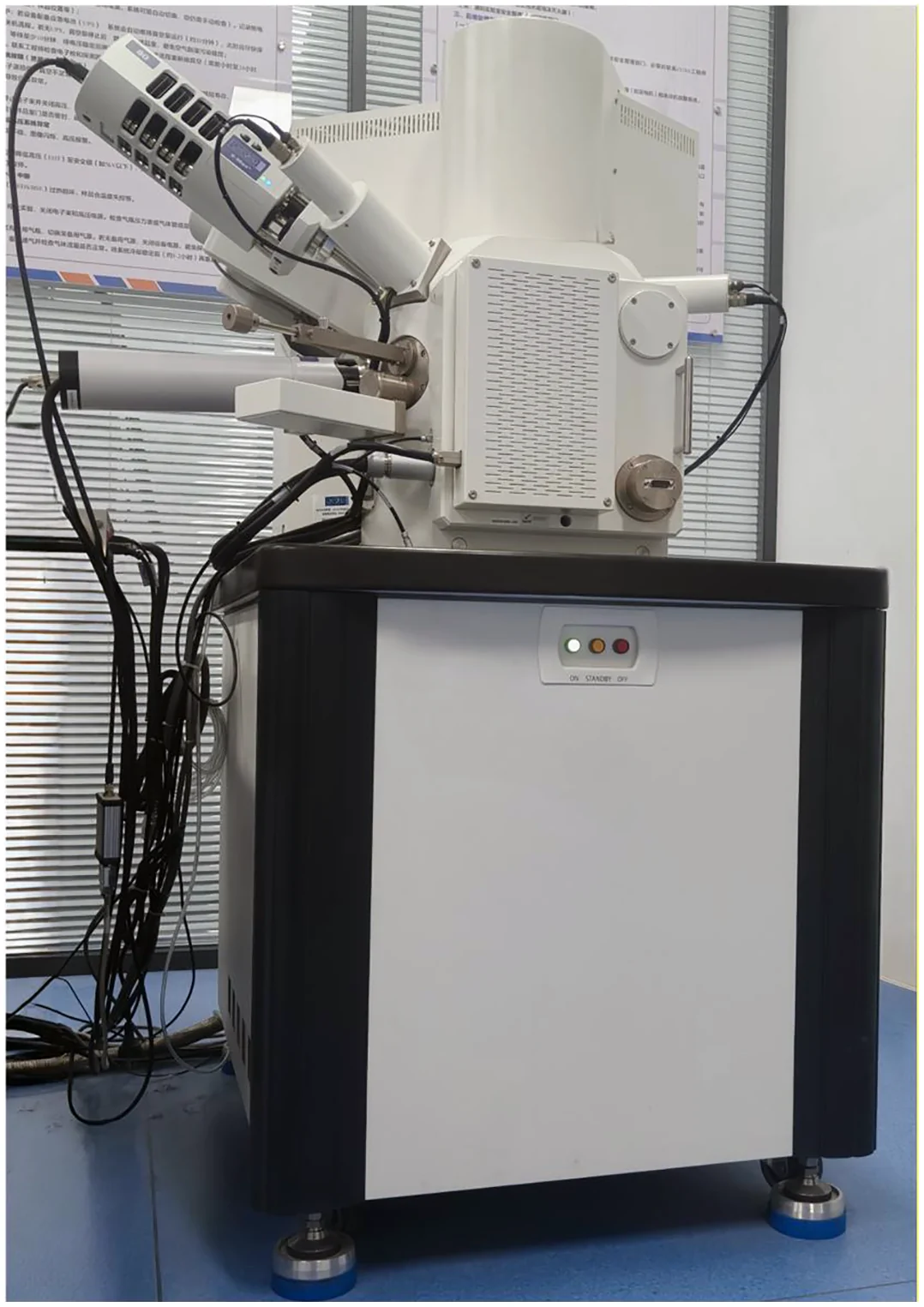
FE-SEM. All photographs are original self-taken images of commercially purchased equipment and consumables, published under a CC-BY 4.0 license.

To evaluate the effects of processing parameters on tensile mechanical properties, the tensile performance of the specimens is measured using an MTS universal material testing machine, as illustrated in Fig 6. Force and displacement data are recorded by the load cell, and the nominal stress–strain curves of the specimens are automatically calculated. To observe the fracture surfaces of tensile specimens, a Zeiss Sigma 500 field emission scanning electron microscope (FE-SEM), as shown in Fig 7, is employed to characterize the cross-sectional morphologies. The fracture mechanisms and related macroscopic behaviors are further analyzed.

## 4. Test plan

### 4.1. Specimen preparation

**Fig 8:**
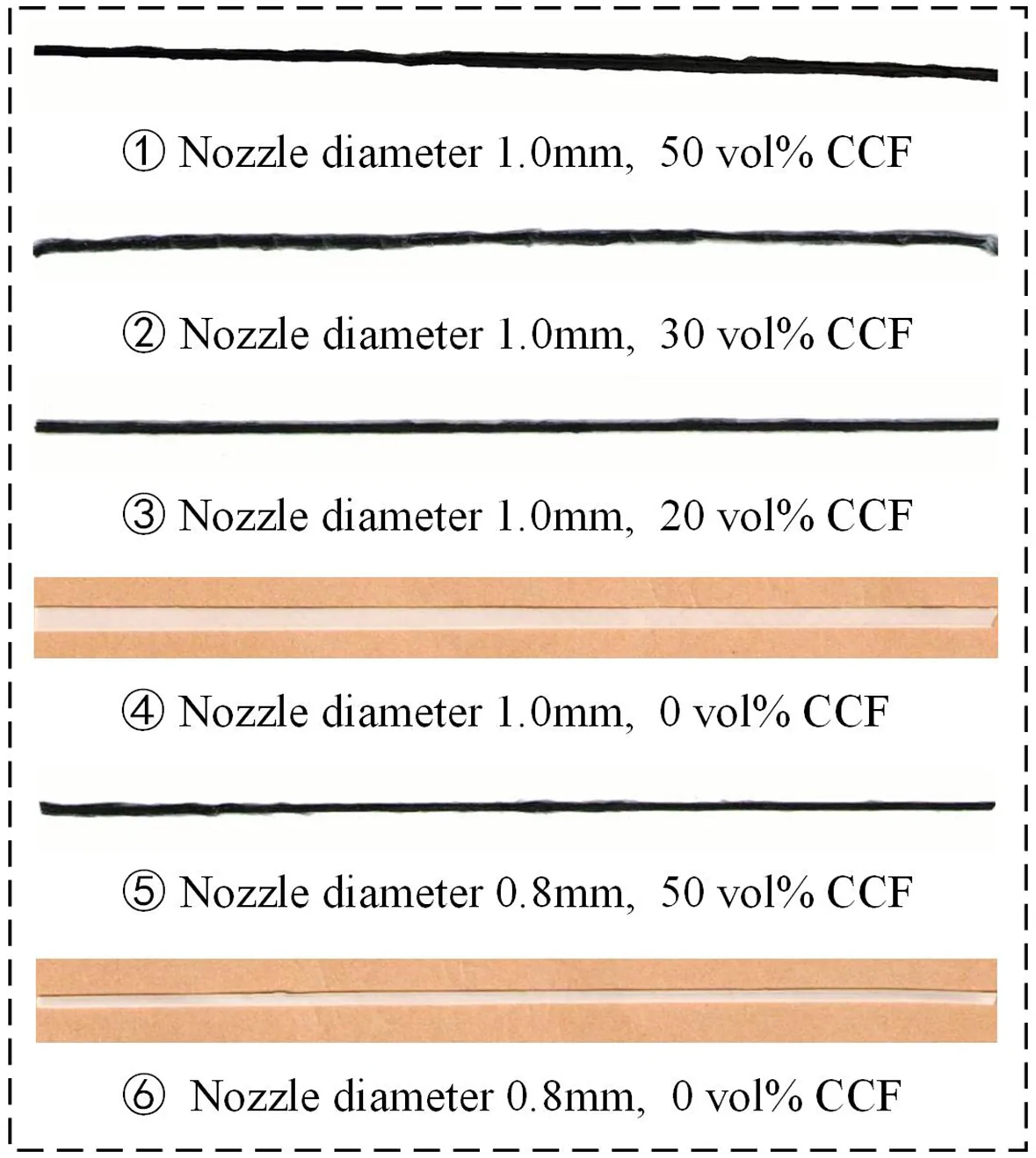
3D-Printed specimen.

In this study, nozzle diameter and CCF volume fraction are selected as the main experimental parameters, with a fixed printing path of 0°. Two nozzle diameters (1.0 mm and 0.8 mm) and four different CCF volume fractions (50%, 30%, 20%, and 0%) are investigated. Combining these processing parameters, a total of six groups of specimens are prepared, as illustrated in Table 1 and Fig 8.

**Table 1:** Specimen types.

Group	Printhead Diameter	CCF volume fraction	Specimen width	Specimen thickness	Specimen length
①	1.0mm	50%	0.2mm	0.3mm	100mm
②	1.0mm	30%	0.15mm	0.3mm	100mm
③	1.0mm	20%	0.15mm	0.3mm	100mm
④	1.0mm	0%	0.1mm	0.3mm	100mm
⑤	0.8mm	50%	0.1mm	0.3mm	100mm
⑥	0.8mm	0%	0.1mm	0.3mm	100mm

### 4.2. Printing process

The printing process was implemented in accordance with the general technical requirements of GB/T 39698−2021 General rules for FDM 3D printing. The fixed printing parameters were set as follows: nozzle temperature 210 °C, hot bed temperature 45 °C, printing speed 40 mm/min, layer height 0.2 mm. During printing, PLA was heated to a viscous flow state inside the nozzle, fully impregnated the continuous carbon fibers, and the integrated CCF/PLA composite filament was extruded and deposited layer by layer on the printing platform to form standard tensile specimens.

### 4.3. Loading and testing methods

**Fig 9:**
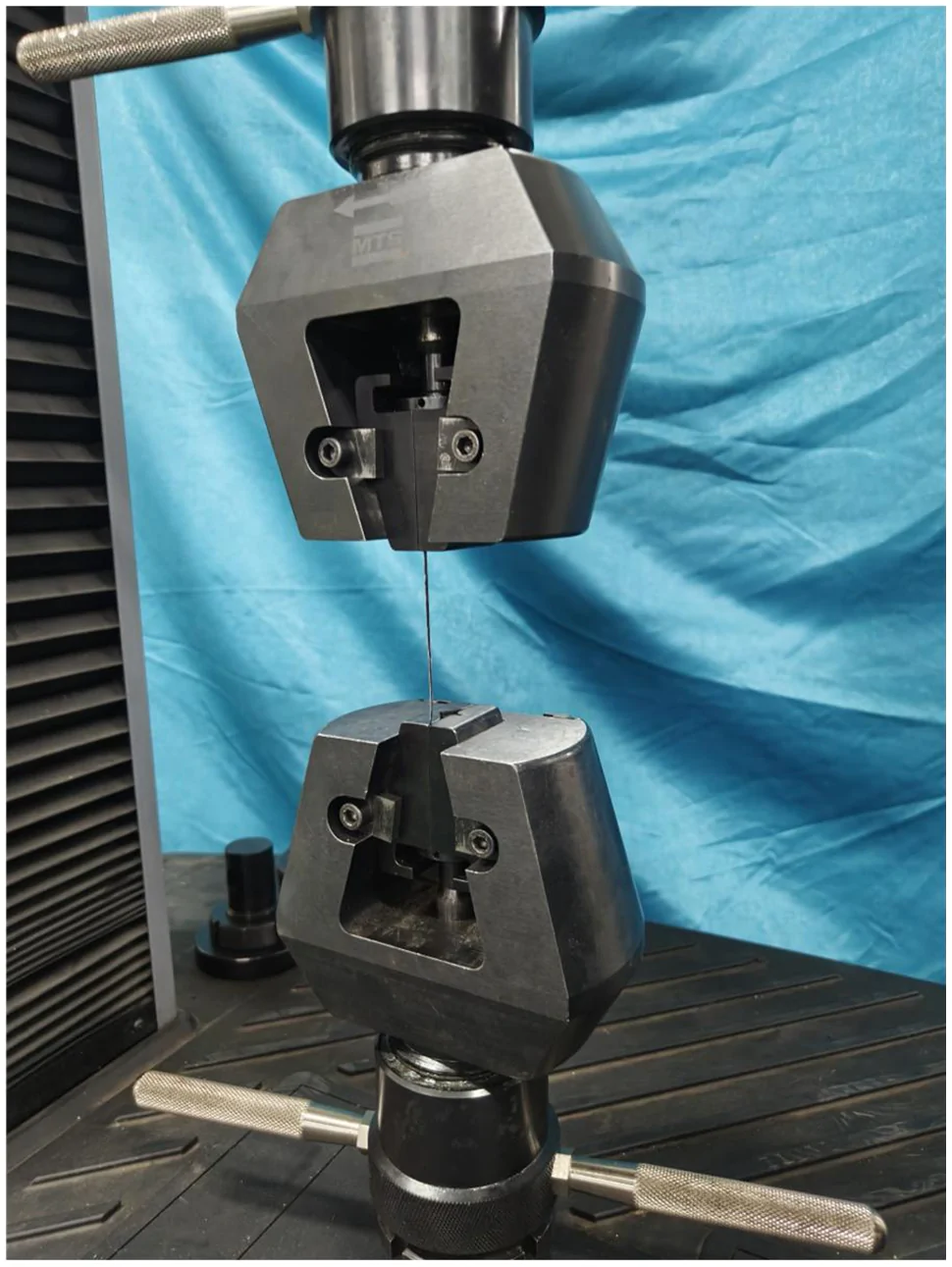
Clamping process.

As shown in Fig 9, the uniaxial tensile test was implemented on an MTS 30 t universal material testing machine in accordance with GB/T 1040.2–2022 under displacement control at a constant loading rate of 2 mm/min. Thin aluminum sheets were attached to both ends of each tensile specimen for reinforcement prior to clamping to avoid local compressive-shear failure at the gripping region induced by excessive clamping force. The load-displacement data were automatically acquired by the load cell, and the nominal stress–strain curves, tensile strength and fracture strain of specimens were calculated synchronously. After tensile testing, typical fracture sections were cut and subjected to gold sputtering to improve electrical conductivity, and their micro-morphologies were characterized using a Zeiss Sigma 500 field emission scanning electron microscope (FE-SEM); the fiber integrity, fiber-matrix interfacial condition, fiber distribution and microscopic failure mechanism of FDM-printed CCF/PLA composites were further analyzed based on the microscopic images.

## 5. Test results

### 5.1. Test results and analysis

(1) Effect of nozzle diameter on stretching performance

Nozzle diameter is a key process parameter that directly determines the extrusion state of composite filaments during FDM printing. Test results show that neat PLA specimens without CCF exhibit obvious plastic deformation under tension regardless of nozzle size, which is inherent to the ductile characteristic of polylactic acid matrix. By contrast, all CCF-reinforced specimens present linear stress–strain responses until fracture with negligible plastic deformation, indicating that the high-stiffness CCF dominates the overall mechanical behavior of composites.

**Fig 10:**
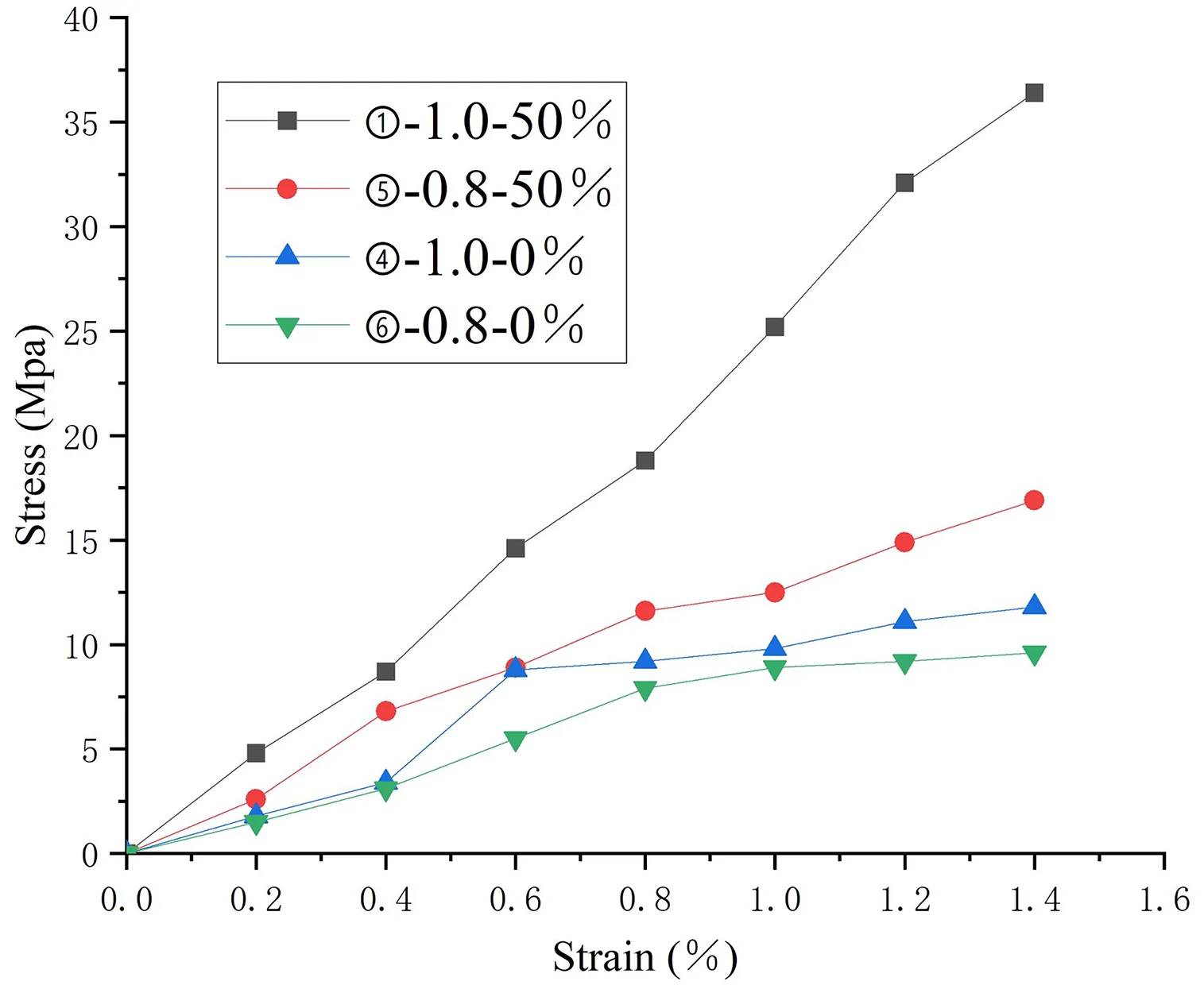
Stress-strain curves of each group of specimens under different nozzle diameters.

According to Fig 10, at an identical CCF volume fraction, specimens printed with the 1.0 mm nozzle possess remarkably higher tensile strength than those fabricated using the 0.8 mm nozzle. The fundamental mechanism lies in the filament extrusion process: the larger-diameter nozzle provides a milder extrusion environment, avoiding excessive squeezing and shearing on continuous carbon fibers. Fibers maintain complete morphological integrity and uniform distribution inside the PLA matrix. In comparison, the narrow 0.8 mm nozzle generates intense extrusion pressure and friction during material output, which easily causes micro-cracks, fiber breakage and disordered arrangement of CCF. Damaged fibers lose their load-bearing capacity, and uneven fiber distribution creates discrete stress concentration regions inside the specimens, jointly leading to the deterioration of tensile performance. Even for pure PLA samples, a larger nozzle also improves tensile strength to a certain extent, as it facilitates full fusion and tighter bonding between adjacent printed layers.

(2) Effect of Carbon Fiber Content on Tensile Properties

**Fig 11:**
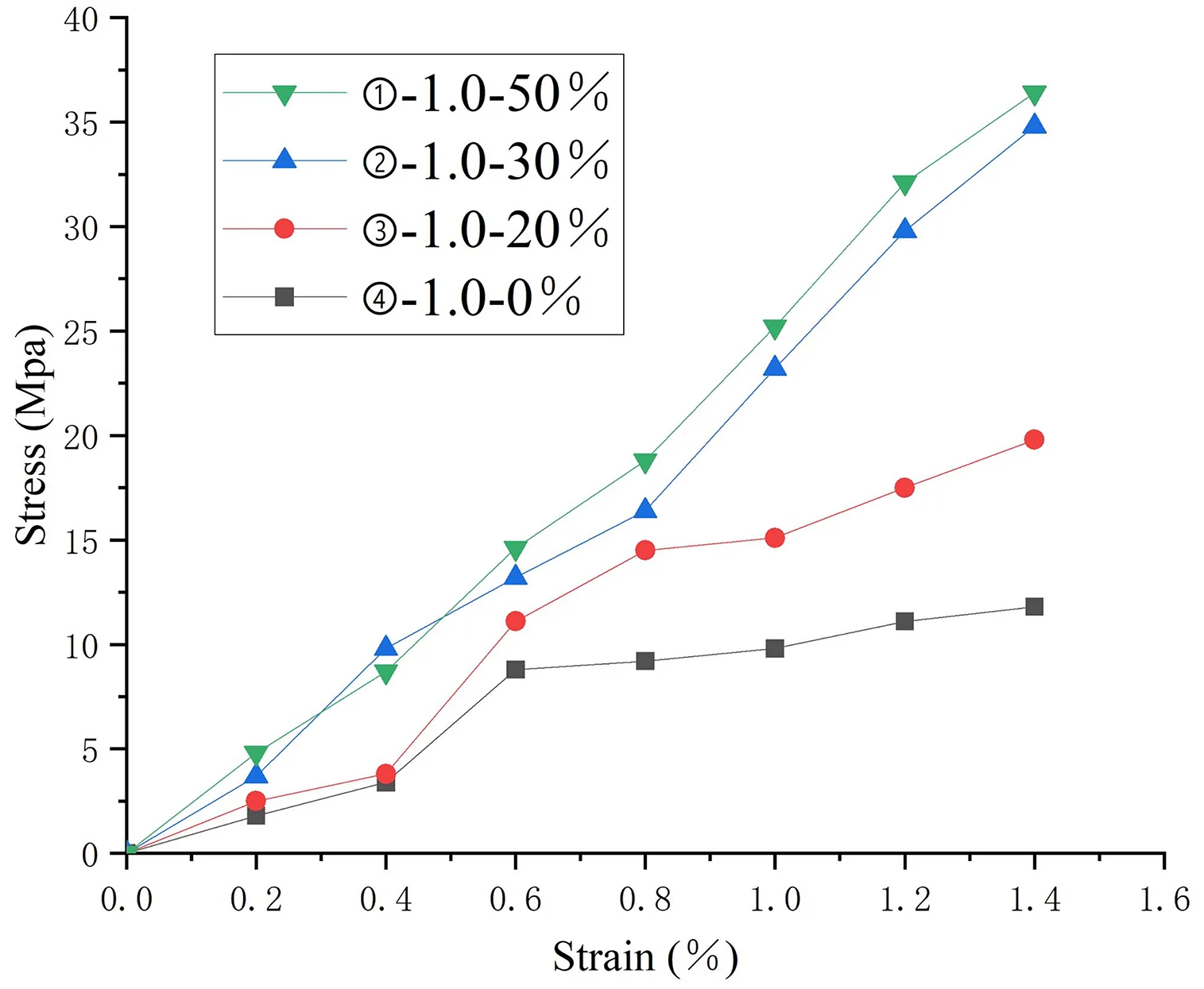
Stress-strain curves of each set of specimens under the same nozzle diameter.

As shown in Fig 11, under the fixed nozzle diameter of 1.0 mm, the tensile properties of composites show an obvious correlation with CCF volume fraction. The tensile strength reaches 36.4 MPa at 50% CCF content, and gradually decreases to 34.8 MPa and 19.8 MPa as the fiber fraction drops to 30% and 20%, respectively. Overall, the tensile strength increases approximately linearly with the rise of CCF volume fraction.

The performance enhancement mechanism of CCF reinforcement is mainly reflected in two aspects. First, CCF has ultra-high specific strength and modulus far exceeding the PLA matrix. After being compounded with PLA, CCF acts as the main load-bearing skeleton inside the composite. When external tensile load is applied, stress is rapidly transferred from the low-strength PLA matrix to high-performance CCF, which bears most of the tensile force and restricts the plastic flow of the matrix. Second, uniformly distributed CCF forms a continuous load-transfer network inside the material. Higher fiber content means denser load-transfer channels, which further improve the overall stiffness and tensile resistance of the composite.

The strain evolution and interfacial failure process can be clearly observed from stress–strain curves. When the tensile strain approaches 0.6%, interfacial debonding occurs between CCF layers and PLA matrix. This phenomenon originates from the mismatched mechanical properties of the two phases: high-rigidity CCF produces tiny strain under tension, while ductile PLA tends to deform greatly. The strain difference induces severe stress concentration at the fiber–matrix interface, destroying the interfacial bonding. After debonding, the synergistic bearing effect of CCF and PLA fails. The PLA matrix independently bears increased load and then cracks. With the continuous propagation of matrix cracks, the load is completely redistributed to CCF layers. Eventually, the CCF skeleton fractures, resulting in the overall failure of the specimen. This also explains why CCF-reinforced composites have no obvious plastic deformation stage.

### 5.2. SEM microfracture morphology

**Fig 12:**
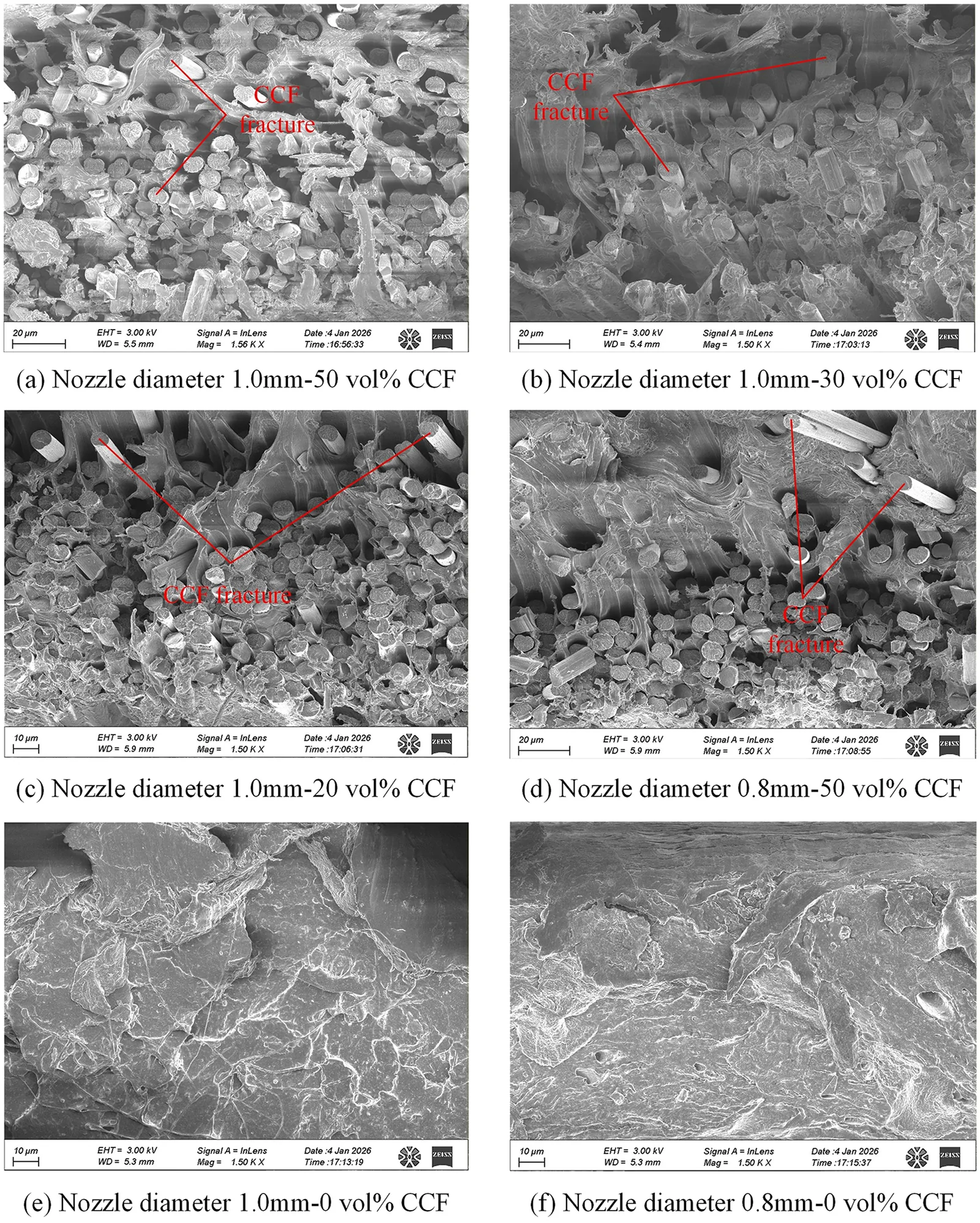
SEM Micro-failure Morphology. (a) Nozzle diameter 1.0 mm-50 vol% CCF. (b) Nozzle diameter 1.0 mm-30 vol% CCF. (c) Nozzle diameter 1.0 mm-20 vol% CCF. (d) Nozzle diameter 0.8 mm-50 vol% CCF. (e) Nozzle diameter 1.0 mm-0 vol% CCF. (f) Nozzle diameter 0.8 mm-0 vol% CCF.

As shown in Fig 12, under the nozzle diameter of 1.0 mm, the fracture surface of the specimen with 50 vol% CCF presents densely and uniformly distributed carbon fibers with intact cross-sections. No obvious fiber pull-out or interfacial debonding is observed, indicating favorable interfacial bonding between fibers and the PLA matrix. External load can be efficiently transferred to carbon fibers, which fracture synchronously, corresponding to the optimal tensile strength and elastic modulus. As the CCF content decreases to 30 vol% and 20 vol%, the fiber distribution density gradually declines and the effective load transfer paths are reduced, leading to deteriorated mechanical properties. The pure PLA specimens only show typical matrix tearing characteristics without any fiber-related features, thus exhibiting the lowest mechanical performance. For specimens filled with 50 vol% CCF, the 1.0 mm nozzle maintains complete fiber morphology, uniform dispersion and tight interfacial bonding, enabling the full exertion of fiber reinforcement. In comparison, the 0.8 mm nozzle causes uneven fiber distribution and fiber damage induced by extrusion, which weakens interfacial bonding and load transfer capacity, and consequently results in a sharp drop in tensile strength. Overall, the SEM microscopic characteristics are highly consistent with the tensile test results. CCF content and nozzle diameter directly affect fiber distribution, structural integrity and interfacial adhesion, and further determine the mechanical properties of composites. Due to the absence of reinforcing fibers, the performance of pure PLA is barely affected by nozzle diameter.

## 6. Discussion on the impact of printing parameters

Combined with mechanical test results and microscopic characterization, the coupling effect of nozzle diameter and CCF volume fraction is discussed from the perspective of practical application. Within the experimental range, increasing nozzle diameter effectively improves the tensile strength of composites. A 1.0 mm nozzle can protect continuous carbon fibers from extrusion damage and guarantee good printing quality, which is more suitable for manufacturing high-performance CCF/PLA parts. The tensile strength of all specimens rises linearly with the increase of CCF volume fraction, owing to the continuous improvement of the internal load-bearing skeleton.

Nevertheless, the performance gain gradually slows down when CCF content increases from 30% to 50%. The difference in tensile strength between the two groups is limited, while the raw material cost and printing difficulty increase significantly. Excessively high CCF content will also reduce the fluidity of composite filaments, easily cause printing blockage, and further deteriorate interfacial bonding. Considering mechanical performance, production cost and process feasibility comprehensively, 30% is determined as the optimal CCF volume fraction for engineering application. In terms of failure characteristics, all CCF-reinforced specimens maintain a maximum tensile strain of approximately 1.4% without plastic deformation, belonging to brittle failure dominated by fiber fracture; pure PLA is characterized by large plastic deformation and ductile fracture. This distinct difference in failure modes provides a reference for the service selection of FDM-printed CCF/PLA composites: fiber-reinforced composites are applicable for load-bearing components requiring high strength and low deformation, while pure PLA is more suitable for non-load-bearing parts with certain toughness requirements.

## 7. Conclusion

In this work, modified FDM equipment was adopted to fabricate CCF/PLA composites with different nozzle diameters and CCF volume fractions, and the regulation mechanism of the two key parameters on tensile properties and fracture behaviors was systematically clarified. The experimental results demonstrate that the 1.0 mm nozzle effectively prevents fiber damage and delivers superior mechanical performance compared with the 0.8 mm nozzle. For specimens printed with the 1.0 mm nozzle, the tensile strengths are 36.4 MPa, 34.8 MPa and 19.8 MPa at CCF volume fractions of 50%, 30% and 20%, respectively, and the tensile strength increases nearly linearly with increasing CCF content. Balancing mechanical performance and production cost, 30% CCF volume fraction is identified as the optimal parameter. FESEM analysis reveals that intact fiber morphology, uniform fiber distribution and strong fiber-matrix interfacial bonding are essential for high tensile performance. Strain mismatch between CCF and PLA induces interfacial debonding, leading to a progressive failure process involving interface separation, matrix cracking and fiber rupture. This study clarifies the coupling mechanism of nozzle size and fiber content, and proposes practical process parameters, which can serve as a reference for FDM manufacturing of carbon fiber composite parts. Further research will focus on multi-mechanical property characterization, multi-parameter optimization, fiber surface modification and environmental resistance evaluation to accelerate the practical application of such composites.

## Supporting information

S1 File Raw stress-strain curve data corresponding to Fig 10 and Fig 11. (ZIP)

S2 File Raw SEM images corresponding to Fig 12. (ZIP)

S3 File Video showing the FDM printing process of CCF/PLA composite materials. (MP4)

S4 File Video showing the tensile testing process of CCF/PLA composite specimens. (MP4)

## References

[1] Chacón JM, Caminero MA, García-Plaza E, Núñez PJ. Additive manufacturing of PLA structures using fused deposition modelling: effect of process parameters on mechanical properties and their optimal selection. Mater Des. 2017;124:143–57.

[2] Morgan AJL, Hidalgo San Jose L, Jamieson WD, Wymant JM, Song B, Stephens P, et al. Simple and versatile 3D printed microfluidics using fused filament fabrication. PLoS One. 2016;11(4):e0152023. doi: 10.1371/journal.pone.0152023 27050661 PMC4822857

[3] Dou H, Cheng YY, Ye WG, Zhang DH, Li JJ, Miao ZJ, et al. Effect of process parameters on tensile mechanical properties of 3D printing continuous carbon fiber-reinforced PLA composites. Materials. 2020;13(17):3850. 32878337 10.3390/ma13173850 PMC7504615

[4] Krajangsawasdi N, Blok LG, Hamerton I, Longana ML, Woods BKS, Ivanov DS. Fused deposition modelling of fibre reinforced polymer composites: a parametric review. J Compos Sci. 2021;5(1):29.

[5] Cheng P, Han Z, Chen Y, Ye L. Recent progress in non-planar 3D printing of continuous fiber-reinforced composites. Composites Part A: Applied Science and Manufacturing. 2025;194:108900. doi: 10.1016/j.compositesa.2025.108900

[6] Kartal F. Mechanical performance optimization in FFF 3D printing using Taguchi design and machine learning approach with PLA /walnut Shell composites filaments. Vinyl Additive Technology. 2025;31(3):622–38. doi: 10.1002/vnl.22195

[7] Ulkir O, Ersoy S. Hybrid experimental–machine learning study on the mechanical behavior of polymer composite structures fabricated via FDM. Polymers. 2025;17:2012. 40808060 10.3390/polym17152012 PMC12349449

[8] Abdi B, Wang Y, Gong H, Su M. Recycling, remanufacturing and applications of semi-long and long carbon fibre from waste composites: a review. Appl Compos Mater (Dordr). 2025;32(4):1237–65. doi: 10.1007/s10443-025-10316-6 40787123 PMC12334481

[9] Latko-Durałek P, Sałasińska K, Bereska B, Bereska A, Czajka-Warowna A, Durałek P, et al. Recycling and reusing of waste aircraft composites in thermoplastic and thermoset matrices. Materials (Basel). 2026;19(3):534. doi: 10.3390/ma19030534 41681222 PMC12898532

[10] Milosan I, Bedo T, Gabor C, Pop MA. Mechanical characteristics of glass-fiber-reinforced polyester composite materials. Materials (Basel). 2025;18(15):3595. doi: 10.3390/ma18153595 40805473 PMC12348993

[11] Zhu WY, Li SX, Long HM, Dong SQ, Wang K, Peng Y. Simulation analysis and optimization of 3D printed continuous carbon fiber reinforced composites. Compos Struct. 2025;355:118840.

[12] Ivanova O, Williams C, Campbell T. Additive manufacturing (AM) and nanotechnology: promises and challenges. Rapid Prototyping Journal. 2013;19(5):353–64. doi: 10.1108/rpj-12-2011-0127

[13] Lou X, Dong L, Tang X, Nan X, Zhang T, Zhao L. Dual-matrix interface strength of 3D printed continuous carbon fiber reinforced composites: Quantitative assessment methodology and critical influencing factors. Additive Manufacturing Letters. 2025;14:100303. doi: 10.1016/j.addlet.2025.100303

[14] Thirugnanasamabandam A, Subramaniyan M, Prabhu B, Ramachandran K. Development and comprehensive investigation on PLA/ carbon fiber reinforced PLA based structurally alternate layered polymer composites. J Ind Eng Chem. 2024;136:248–57.

[15] Jatti VS, Sapre MS, Jatti AV, Khedkar NK, Jatti VS. Mechanical properties of 3D-printed components using fused deposition modeling: optimization using the desirability approach and machine learning regressor. ASI. 2022;5(6):112. doi: 10.3390/asi5060112

[16] Li NY, Li YG, Liu ST. Rapid prototyping of continuous carbon fiber reinforced polylactic acid composites by 3D printing. J Mater Process Technol. 2016;238:218–25.

[17] Tian X, Liu T, Yang C, Wang Q, Li D. Interface and performance of 3D printed continuous carbon fiber reinforced PLA composites. Composites Part A: Applied Science and Manufacturing. 2016;88:198–205. doi: 10.1016/j.compositesa.2016.05.032

[18] Wang XY, Liu TR, Lü XZ, Wang GS, Yue JG. Experimental study on tensile mechanical properties of 3D printed continuous carbon fiber composites. Journal of Mechanical Engineering. 2025;61(11):396–405.

[19] Araya-Calvo M, López-Gómez I, Chamberlain-Simon N, León-Salazar JL, Guillén-Girón T, Corrales-Cordero JS. Evaluation of compressive and flexural properties of continuous fiber fabrication additive manufacturing technology. Addit Manuf. 2018;22:157–64.

[20] Chen CF, Shao MW, Liu K, Zhong WW, Djassi I, Wang AD. Enhanced thermal and electrical properties of photosensitive resin matrix composites with hexagonal boron nitride nanosheets. J Inorg Organomet Polym Mater. 2023;33:319–27.

[21] Rajpurohit SR, Dave HK. Effect of process parameters on tensile strength of FDM printed PLA part. RPJ. 2018;24(8):1317–24. doi: 10.1108/rpj-06-2017-0134

[22] Kechagias JD, Vidakis N, Petousis M. Parameter effects and process modeling of FFF-TPU mechanical response. Mater Manuf Process. 2023;38:341–51.

[23] Elmrabet N, Siegkas P. Dimensional considerations on the mechanical properties of 3D printed polymer parts. Polym Test. 2020;90:106656.

[24] Lou X, Zhao L, Gao Y, Nan X. 3D-printed electrode/electrolyte architectures for high-performance lithium-ion batteries: mechanisms, materials, and challenges. ACS Omega. 2025;10(27):28630–42. doi: 10.1021/acsomega.5c02247 40687034 PMC12268461

[25] Kumar M, Ranvijay R, Vashishtha G, Chauhan S, Rangappa SM, Siengchin S. On novel sandwiching of agave sisalana waste into polylactic acid by machine learning-assisted 3D printing for manufacturing sustainable structures. JMEPEG. 2026;35:13130–44.

[26] Kumar M, Ayyappan V, Kumar R, Singh MK, Rangappa SM, Siengchin S. Performance of strategically interleaved continuous sisal fiber core 3D printed PLA composites for engineering applications. Int J Adv Manuf Technol. 2026;143:3601–16.

[27] Rana M, Kumar M, Kumar R. Fused deposition modelling approach in recycled polypropylene/aluminum powder composites for sustainable development. Appl Sci Eng Prog. 2024;17(4):7501.

[28] Kumar M, Siengchin S, Singh J, Arora G, Kumar R, Rangappa SM. Recent progress in 5D printing: processes, materials, applications, and future trends. Adv Mater Technol. 2026;11(e01337).

[29] Girija M, Sampath Kumar T. State of art on evaluation of three- to six-dimensional novel additive manufacturing technology for biomedical applications. Proceedings of the Institution of Mechanical Engineers, Part E: Journal of Process Mechanical Engineering. 2024;240(3):3580–601. doi: 10.1177/09544089241281985

[30] Girija M, Sampath Kumar T. Characterization of akermanite (AKT) and zirconia-infused PMMA bone cement composite with superior physicochemical, mechanical, and bioactive properties for enhanced orthopaedic performance. Composite Interfaces. 2024;32(6):817–41. doi: 10.1080/09276440.2024.2442169

[31] Girija M, Sampath Kumar T, Naher S. Synergistic akermanite/PMMA composites with nature inspired structure via 3D printing for orthopaedics. J Mater Res Technol. 2025;39:5238–50.

