# Supplementary material for: Effects of nozzle diameter and fiber volume fraction on tensile properties of FDM-printed CCF/PLA composites
Source: PLoS One. 2026 Aug 19;21(8):e0356177. doi: 10.1371/journal.pone.0356177 (PMC13489468; doi:10.1371/journal.pone.0356177)
Supplement: Supplementary file 1 [file pone.0356177.s001.zip › S1_Stress-strain curve/Nozzle diameter 1.0mm-0 vol% CCF.pdf]

# Default Trial Run Report

| | |
|---|---|
| project name | project 1 |
| user name | MTS |
| Test Name | MTS EM TensileTest – MyTensileTest-5-19 40 |
| Trial Operation Name | Test Run 6 |
| Trial Operation Date | 2025/12/19 |

TestOperationRecheckCurve

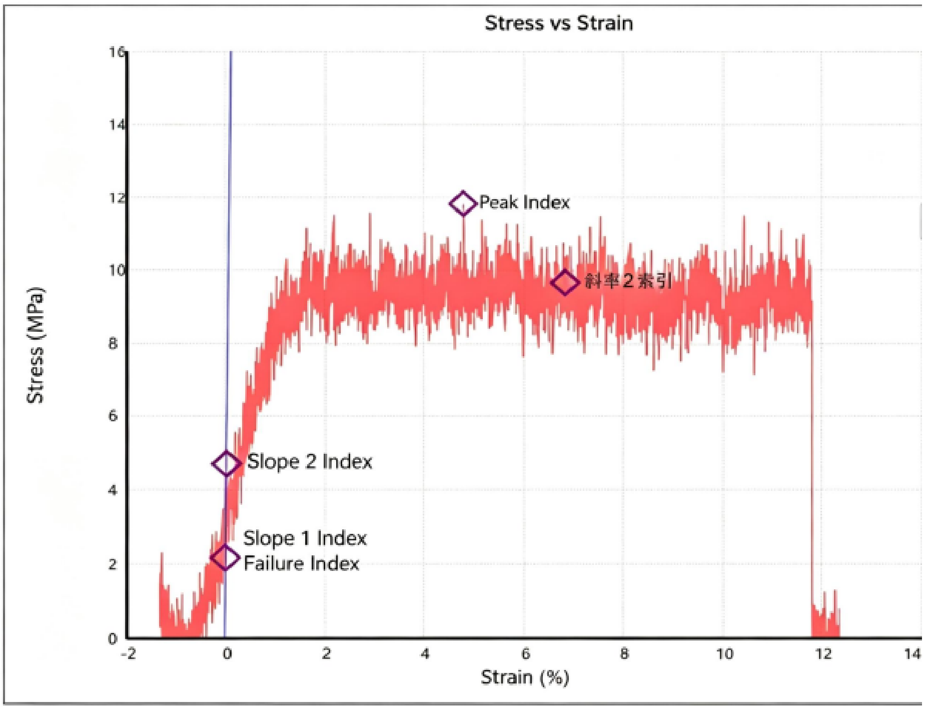


| Display name | Price | Unit |
|---|---|---|
| Test run | The test has been stopped | |
| Extender | 1.000 | mm |
| Peak stress | 11.8 | MPa |
| Peak load | 9.278 | N |
| Strain at the time of failure | 6.817 | % |
| Diameter | 1.000 | mm |
