# Supplementary material for: Effects of nozzle diameter and fiber volume fraction on tensile properties of FDM-printed CCF/PLA composites
Source: PLoS One. 2026 Aug 19;21(8):e0356177. doi: 10.1371/journal.pone.0356177 (PMC13489468; doi:10.1371/journal.pone.0356177)
Supplement: Supplementary file 1 [file pone.0356177.s001.zip › S1_Stress-strain curve/Nozzle diameter 1.0mm-30 vol% CCF.pdf]

Default Trial Run Report

project name	project 1
user name	MTS
Test Name	MTS EM TensileTest – MyTensileTest-5-19 40
Trial Operation Name	Test Run 6
Trial Operation Date	2025/12/19

TestOperationRecheckCurve

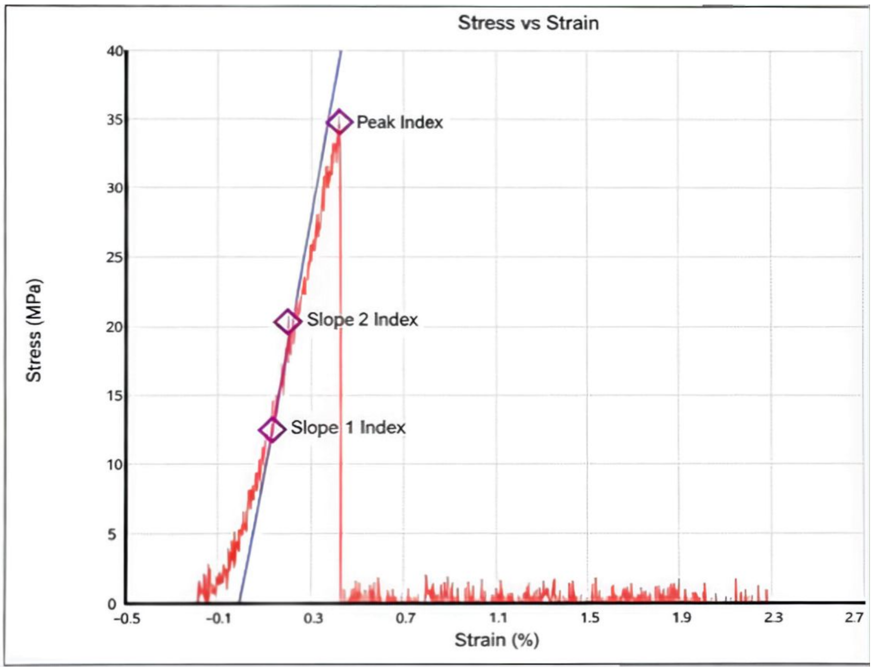

Display name	Price	Unit
Test run	The test has been stopped	
Extender	1.000	mm
Peak stress	34.8	MPa
Peak load	27.329	N
Strain at the time of failure	0.424	%
Diameter	1.000	mm
